# deltaXpress (ΔXpress): a tool for mapping differentially correlated genes using single-cell qPCR data

**DOI:** 10.1186/s12859-023-05541-4

**Published:** 2023-10-27

**Authors:** Alexis Germán Murillo Carrasco, Tatiane Katsue Furuya, Miyuki Uno, Tharcisio Citrangulo Tortelli, Roger Chammas

**Affiliations:** 1 Center for Translational Research in Oncology (LIM24), Instituto Do Cancer Do Estado de Sao Paulo (ICESP), Hospital das Clinicas da Faculdade de Medicina da Universidade de Sao Paulo (HCFMUSP), São Paulo, SP CEP 01246-000 Brazil; 2 Comprehensive Center for Precision Oncology, Universidade de Sao Paulo, São Paulo, Brazil

**Keywords:** Gene expression, qPCR, Shiny, Gene correlation, Single-cell analysis

## Abstract

**Background:**

High-throughput experiments provide deep insight into the molecular biology of different species, but more tools need to be developed to handle this type of data. At the transcriptomics level, quantitative Polymerase Chain Reaction technology (qPCR) can be affordably adapted to produce high-throughput results through a single-cell approach. In addition to comparative expression profiles between groups, single-cell approaches allow us to evaluate and propose new dependency relationships among markers. However, this alternative has not been explored before for large-scale qPCR-based experiments.

**Results:**

Herein, we present deltaXpress (ΔXpress), a web app for analyzing data from single-cell qPCR experiments using a combination of HTML and R programming languages in a friendly environment. This application uses cycle threshold (Ct) values and categorical information for each sample as input, allowing the best pair of housekeeping genes to be chosen to normalize the expression of target genes. ΔXpress emulates a bulk analysis by observing differentially expressed genes, but in addition, it allows the discovery of pairwise genes differentially correlated when comparing two experimental conditions. Researchers can download normalized data or use subsequent modules to map differentially correlated genes, perform conventional comparisons between experimental groups, obtain additional information about their genes (gene glossary), and generate ready-to-publication images (600 dots per inch).

**Conclusions:**

ΔXpress web app is freely available to non-commercial users at https://alexismurillo.shinyapps.io/dXpress/ and can be used for different experiments in all technologies involving qPCR with at least one housekeeping region.

**Supplementary Information:**

The online version contains supplementary material available at 10.1186/s12859-023-05541-4.

## Background

Currently, the scientific community has been migrating towards high-throughput technologies that generate large volumes of data [1, 2]. Thus, many efforts have focused on analyzing gene expression data from Next Generation Sequencing (NGS) or microarray platforms [3, 4]. Nevertheless, these data need to be validated through more specific techniques such as quantitative PCR (qPCR). Through this method, researchers can assess differentially expressed genes between two or more groups using a conventional approach. In this type of analysis, many tested genes could be excluded because of the absence of statistical differences in the comparison of their expression between the experimental groups, leaving aside the possible interactions that these genes may have with others in different cellular contexts.

**Fig. 1:**
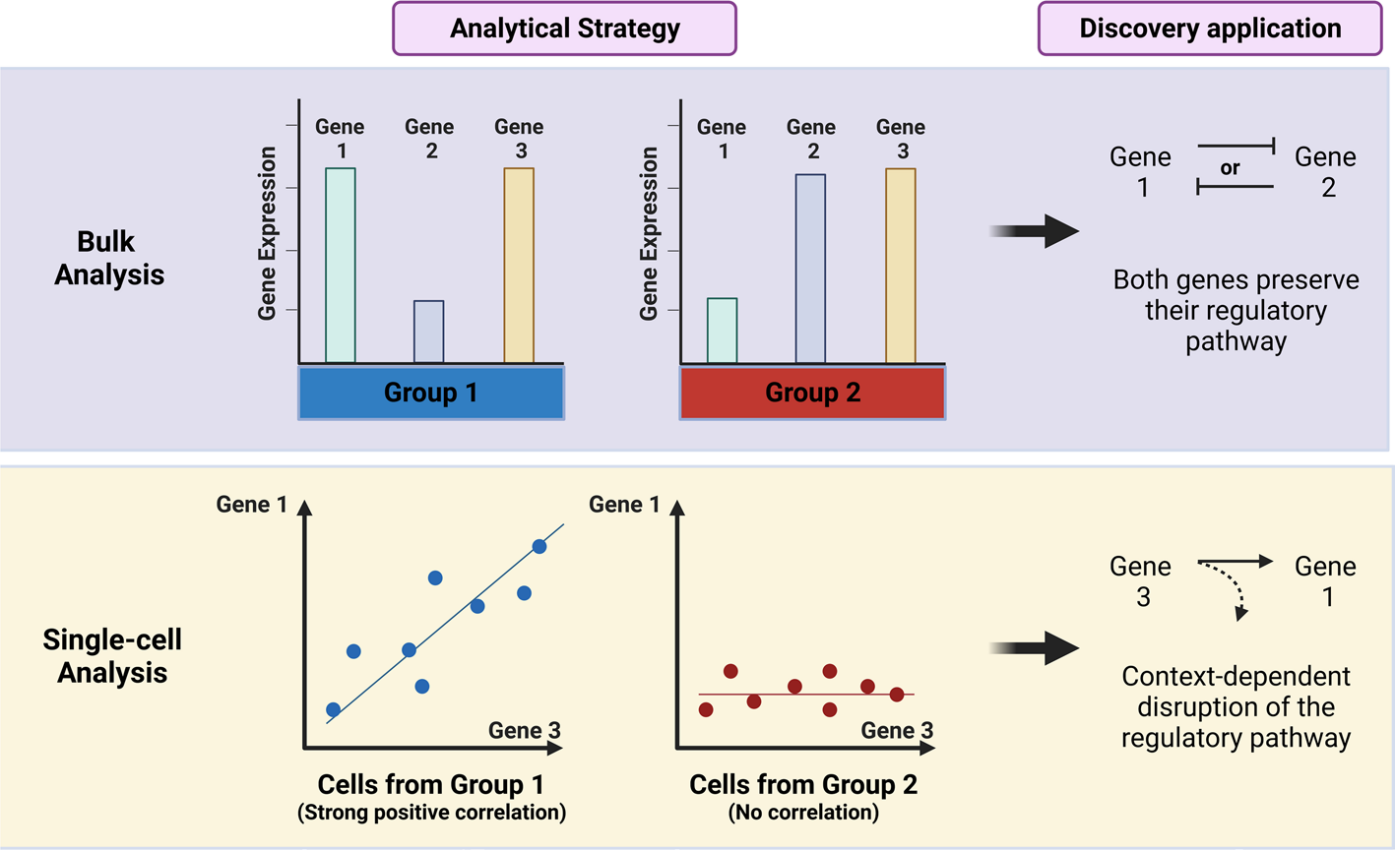
The ΔXpress app can be applied for proposing markers in bulk and single-cell qPCR-based experiments. The conventional comparative analysis of gene expression levels enables to find deregulated genes and propose possible regulatory pathways. In addition, changes in the correlation profile between two genes would provide insights regarding a potential context-dependent disruption of regulatory pathways. Single-cell or large-scale bulk approaches can be used to represent both scenarios to increase our discovery applications

After the description of tumor rewiring [5, 6], there is a need to study differentially correlated genes, a term involving pairwise genes that change their correlation profiles according to different conditions, for example, biological or experimental treatments vs. control samples. Large volumes of data are required to run a robust correlation analysis, which is complicated to obtain using conventional bulk experiments. Thereby, new research initiatives have proposed single-cell or large-scale experiments to understand the behavior of these genes among themselves. According to this hypothesis, by finding differentially correlated genes, researchers could describe additional targets for context-dependent regulatory pathways (Fig. 1).

Recently, researchers have used qPCR strategies for performing single-cell experiments [7–9]. Here arises the need to implement reproducible and easy-to-use tools to normalize these data to obtain quick comparisons with additional information to answer the research hypotheses and to have the advantage of generating the graphics required for publication. Though some web-based applications have been published to analyze qPCR data [10–13], we supplement conventional analyses with perspective opportunities given by single-cell or large-scale techniques: the analysis of differentially correlated genes.

For the ΔXpress application, besides conventional strategies for comparing expression levels between groups, the analysis of co-dependent genes through a differentially correlated profile would be very helpful and relevant for understanding different mechanisms of gene regulation. We envisage that this strategy would allow researchers to find evidence of gene disruption, as it has been proposed for diseases such as cancer [6, 14], which results in direct or indirect effects on pairwise genes to aggregate information on genetic profiles for a determined group.

## Implementation

The ΔXpress is a web application produced for analyzing qPCR data using cycle threshold (Ct) values as input. It allows the processing of a large volume of data in a few minutes, for example, as provided by single-cell experiments. This application was developed using the R software v.4.3.0 with the following packages: shiny (v.1.7.4), readxl (v.1.4.2), tidyr (v.1.3.0), tidyverse (v.2.0.0), dplyr (v.1.1.2), ggplot2 (v.3.4.2), ggpubr (v.0.6.0), scales (v.1.2.1), EnhancedVolcano (v.1.18.0), Hmisc (v.5.1–0), plotly (v.4.10.2), biomaRt (v.2.56.1), and shinycssloaders (v.1.0.0). Once this application is stored on the Shinyapps server, it does not require any software besides your browser.

**Fig. 2:**
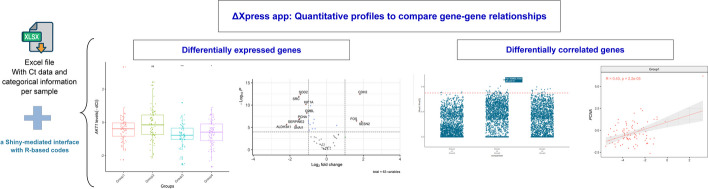
Representation of the main results produced by the ΔXpress app with custom data. After loading the spreadsheet containing your Ct values in the web app, you can normalize your data and perform: (1) conventional comparisons such as boxplots to show the expression of a gene per group and volcano plots, or (2) innovative comparisons such as dot plots to show differentially correlated genes between pairwise groups and scatter plots

This app is based on seven modules: Input, Data Normalization, Expression Analysis, Volcano Plots, Correlation Analysis, Scatter Plots, and Glossary (Fig. 2). The Input module allows the researcher to upload a .txt, .csv, .xls or .xlsx-format file containing a first column with sample names, a second column with a group classification, and the following columns with the Ct values for all evaluated genes (including housekeeping genes).

After loading the data, it is possible to use the Data Normalization module to identify the more stable housekeeping genes within the set of samples analyzed. The system will choose the best pair of genes for data normalization using the stability values for each gene provided by the NormFinder algorithm [15]. Then a table with the normalized data can be downloaded in comma-separated value (CSV) format. The analytic strategies will continue in one of the following four modules, or the researcher will choose to learn more about their genes in the Glossary module.

### Data normalization

The ΔXpress application uses a customized edition of the NormFinder algorithm [15] to normalize expression levels (Ct values). Unlike their original version, we edited the NormFinder algorithm for only processing Ct values from samples belonging to experimental groups (second column of the input table). The system can detect technical replicates using the same identifiers in the first (sample name) and second (primary group name) columns. If the system detects two Ct values for a technical replicate, a geometric mean Ct value will be used for the next steps. After evaluating Ct values, the NormFinder algorithm will show two gene lists (single and paired) with their respective stability values. Lower stability values mean more stable gene(s). With this observation, the system will use the best pair of genes to calculate the mean value per sample and normalize all genes using the Livak method [16]. It is essential to mention that the Livak equation assumes the PCR reaction primer efficiencies are similar (usually between 90 and 110%) between the genes of interest and the housekeeping genes. This efficiency is usually already checked in the design of primers in large-scale (or single-cell) qPCR or must be checked manually before entering the data in the ΔXpress application. Otherwise, the Pfaffl equation [17] accounts for any efficiency differences and must be used by including quantification cycle values for a calibrator sample run along with samples in the analysis. In this version, the ΔXpress app does not support the Pfaffl equation.

As an additional and customizable feature, which is restricted to researchers inputting a .xlsx (or .xls) file, the system can accept a user-selected list of housekeeping genes to perform this normalization. This alternative must be applied if your analysis only includes one housekeeping region. After data normalization, all subsequent modules automatically receive the table containing the sample names, their main classification, and normalized expression (−ΔCt) of all genes.

### Expression analysis

In this module, the ΔXpress app will emulate a bulk analysis for comparing gene expression levels between experimental groups. After running the expression analysis, the system will filter the selected gene and check the data for normal distribution for each group using the Kolmogorov–Smirnov test. The *p* values for each group will be displayed on the “Normality” tab. According to the data distribution, the system will automatically choose the proper (parametric or non-parametric) test to compare means between groups. The system uses the Mann–Whitney test (non-parametric) or Student t-test (parametric) to estimate *p* values between two groups. Additionally, the system will display the name of pairwise groups being compared, the number of samples for each group, the median expression level, and the fold change between groups in the “Results” tab. Since this analysis is limited to pairwise comparisons, the reference group can be customized. Finally, this module uses two boxplot schemes to graphically show differences in the expression levels in the selected genes between all groups. The first boxplot shows the current -ΔCt values, whereas the second plot calculates this data in relation to a specific group using the relative expression analysis 2^(−ΔΔCt)^ [16]. In this last plot, the user-selected reference group will have its median value of 2^(−ΔΔCt)^ equal to 1. In both plots, the statistical significance will be represented by asterisk format: *p* ≥ 0.05 (ns), *p* < 0.05 (*), *p* ≤ 0.01 (**), *p* ≤ 0.001 (***), and *p* ≤ 0.0001 (****).

### Volcano plots

For this module, the system has been configured to filter normalized data for all genes in two user-selected groups. After, all genes will be compared using parametric or non-parametric tests (depending on the user’s preferences). With these results, a table will be built containing information about the number of samples for each group, the median level, fold change, and *p* value for each gene. Then, *p* values will be adjusted using the Benjamini–Hochberg method [18]. This table will be displayed on the “Table” tab, while a volcano diagram will be shown on the “Graphs” tab. For the volcano plot, it is possible to choose for adjusted or unadjusted *p* values as well as fold change and *p* value thresholds. The volcano plot displays labels for featured genes based on fold change and *p* value parameters.

### Correlation analysis

In the Correlation Analysis module, the normalized data will be divided according to pairwise groups. For each pairwise group comparison, the system will create a matrix. Then the system will use its algorithm to estimate the correlation coefficients and *p* values between gene pairs based on Spearman or Pearson correlation (according to the data distribution). Given the user-defined *p* value threshold, all *p* values above that threshold will be converted to zero once a non-significant *p* value shows no correlation regardless of its correlation coefficient (R-value). Then these values will be operated using the next equation:

$$\text{dR-val=|}{\text{R value}}_{\text{Group 1}}-{\text{R value}}_{\text{Group 2}}\text{|}$$

Therefore, for each pair of genes, the system will produce $\frac{\mathrm{n}*(\mathrm{n}-1)}{2}$ values in which “n” is the number of groups. The result of the cited equation represents the dR-val and ranges from 0 to 2. If the dR-val is equal to 0, there is no alteration in the correlation profile, but if this value is equal to 2, it means a complete alteration in the correlation profile. A completely altered correlation profile represents the transition of pairwise genes from strong positive to strong negative correlation (or vice versa) between two experimental groups. All values will be plotted in a dynamic dot plot showing all combinations involving a user-defined group. Then, it is possible to pass the mouse cursor over one of those points to know the value of the equation and the pair of genes involved.

### Scatter plots

The Scatter Plots module will filter normalized data by selecting only two groups and two genes defined by the user. The filtered data will be displayed in an XY plot (with each gene represented in an axis). This graph will be divided into two fields (one for each selected group). Each plot field includes a trend line with r and p values from correlation analysis.

### Glossary

This module will collect gene identifiers (Gene Name, Ensembl Gene ID, Ensembl Transcript ID, or NCBI Entrez ID) from the input table. Then, authors must indicate the proper format of gene identifiers and the corresponding species. After obtaining the latest available information, the system will search for complementary information such as the genome version of the database, Entrez ID, Gene Name, and Gene Description using the Ensembl database [19]. In addition, the system will retrieve external links to GeneCards [20], GenBank [21], and Ensembl sources. Currently, the ΔXpress app supports information for human, rat, mouse, and *D. melanogaster* species. However, additional species can be added at the request of research groups, even for less studied species with an appropriate NCBI/Ensembl annotation.

## Results and user-guide

### Example file

To test the ΔXpress application, we run a single-cell experiment to produce a data set of expression levels of 68 genes in 328 samples distributed in four main groups: Group1, Group2, Group3, and Group4. The list of 68 genes includes five housekeeping genes identified as A (*ACTB*), B (*B2M*), C (*GAPDH*), D (*GUSB*), and E (*HPRT1*). You can download a zipped folder from the Additional file 1 and choose one of the files for running the app. Please note that this spreadsheet includes some missing values as blank cells.

### Inputting files

**Fig. 3:**
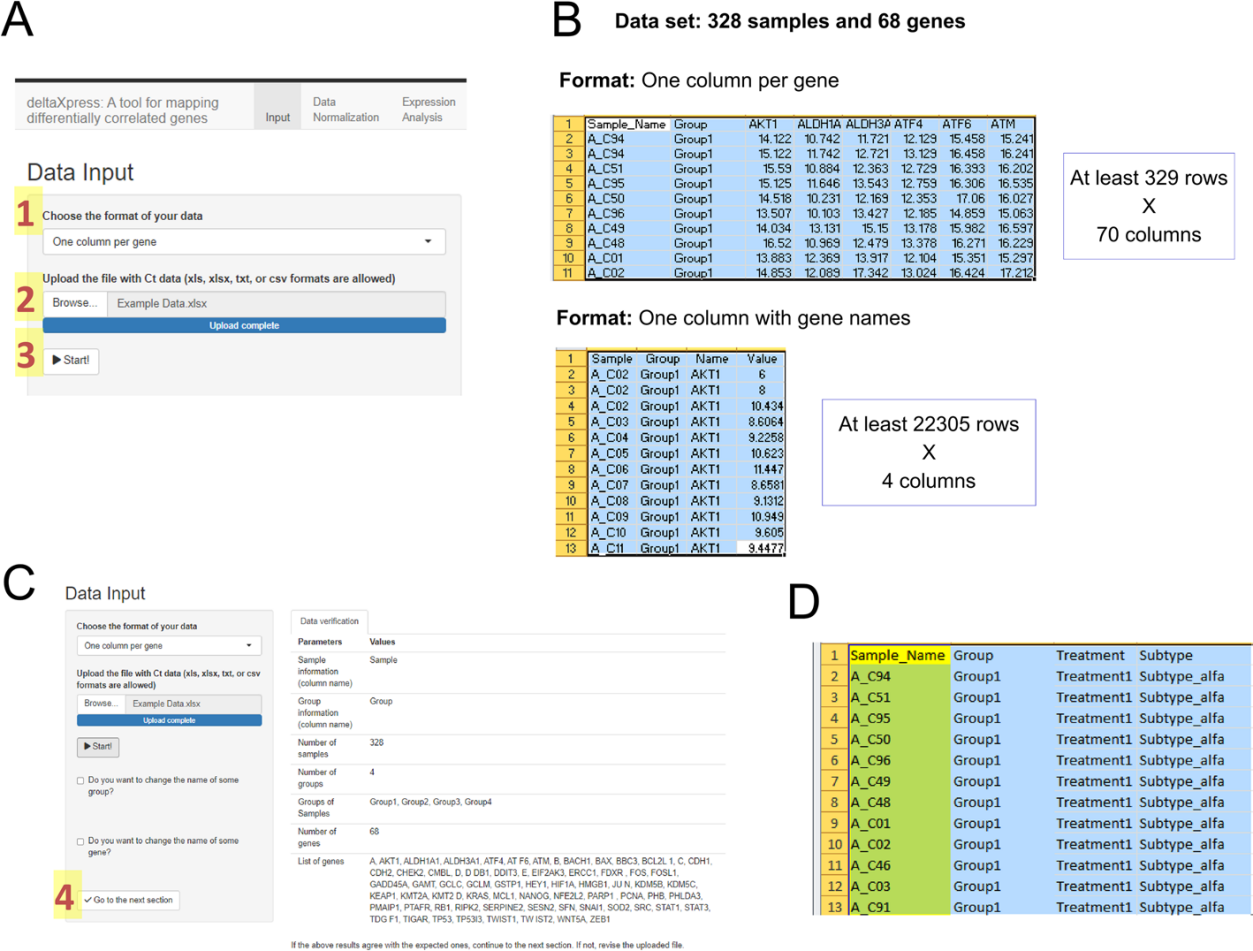
Data Input module. (**A**) Visualization of the first module of the ΔXpress application before loading the input file. To input files, we need to define the format (1), select the file from our directory (2), and click on Start! (3). The ΔXpress app accepts four data formats (**B**). You can use these examples to define the format of your data. After uploading the input file, the ΔXpress app will display a summary of the data (**C**). If you agree with this information, you must click “Go to the next section” (4). Optionally, for researchers inputting Excel files, you can include a second sheet with additional information for each sample (**D**). You can use this space to add sample data to compare with gene expression in subsequent modules. Once you include information for all samples (identified in the first column), the system will automatically recognize it

The ΔXpress application accepts a .txt, .csv, .xls, or .xlsx-format file (Fig. 3A) that includes two mandatory columns (sample and group names) with their corresponding Ct values for all analyzed genes. To record Ct values, the ΔXpress application accepts two commonly used formats (Fig. 3B): a large table with only four columns (sample name, group name, gene name, and Ct values) and a table with an indeterminate number of columns (sample name, group name, and a column per gene). After selecting the proper format for your data, you need to upload the file and click "Start" to read it. If the data from the file matches the format selected in the app, the system will retrieve the following message “File correctly loaded. Please, click on Start!”. Otherwise, the system will display “Please, verify the format of the input file.”. Next, the system will retrieve a summary table that displays information about your data (Fig. 3C).

You must check if all the data was recorded correctly. If the data is correct, you can go to the next section. Otherwise, you can change the name of some gene/group or revise the input file. In our example file, the housekeeping genes were identified as A, B, C, D, and E. We can then use the option to rename genes by replacing A for *ACTB*, for instance. Our dataset has 328 samples, so we expect 329 rows in the file with one column per gene (including the row with column names). However, the input file can have more rows if technical replicas are included. If you include technical replicas, be sure that all rows of the same sample have the same name in the first column. For example, "A_C94", instead of "A_C94_1", "A_C94_2", etc.

Optionally, for researchers inputting Excel files, it is possible to include a second sheet with secondary characteristics of your data (Fig. 3D). To match these data with the primary data (first sheet of the input file), you must keep one identifier per sample in the first column. Starting in the second column, you can add information as one column per category.

### Data normalization

**Fig. 4:**
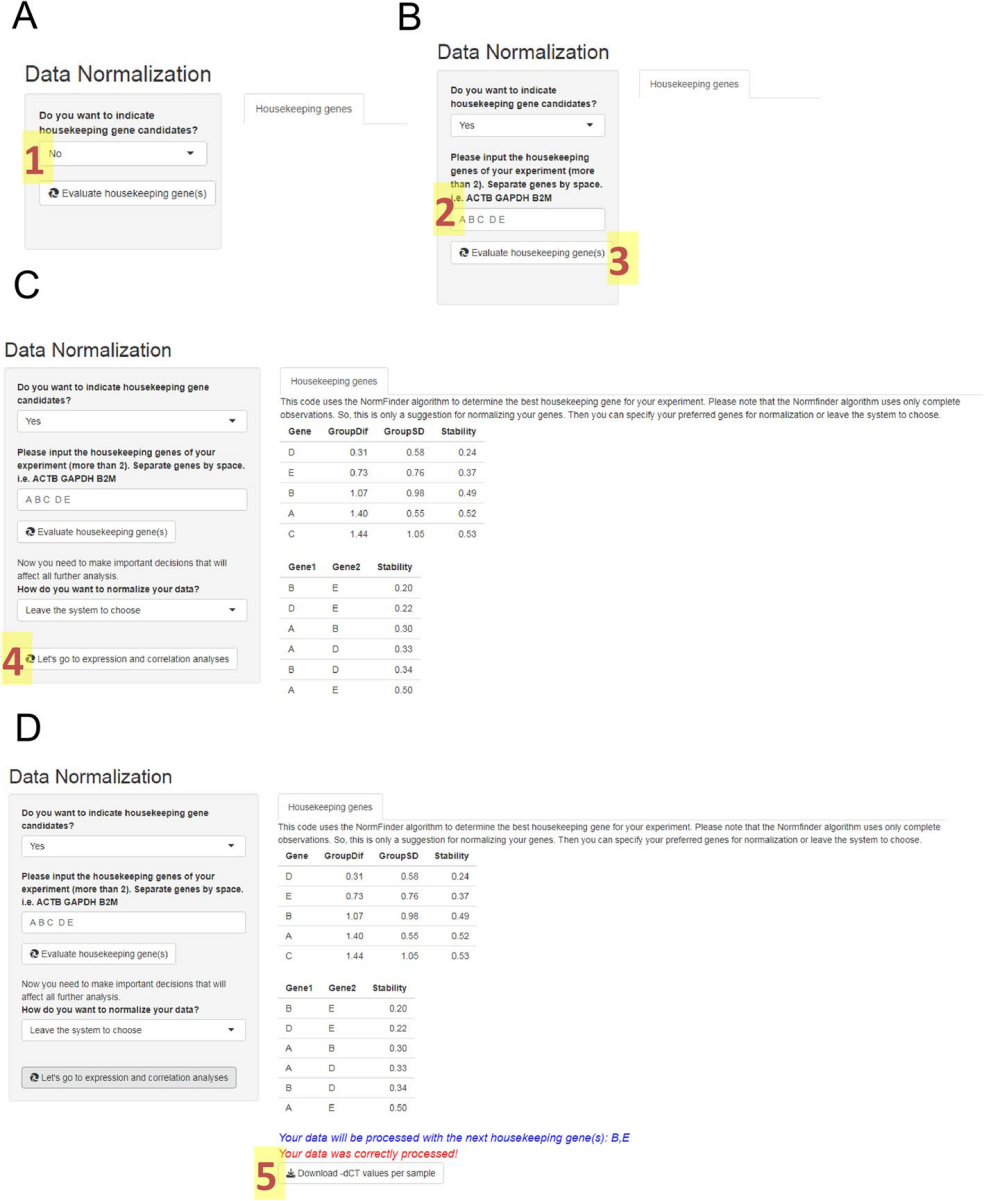
Data Normalization module. After entering the data, it is recommended to indicate the list of housekeeping genes that you used (**A**). You must indicate that you have a list of genes (1), and new question bars will be opened (**B**). Here you should describe all candidate housekeeping genes separated by spaces (2) and request their evaluation (3). After observing the stability values (**C**), you can request a default normalization of your data using the best combination of housekeeping genes (4). Since you have normalized your data (**D**), you can download a table with the normalized −ΔCt values (5) or move on to the next modules

After loading the data, you can identify the putative housekeeping genes you used in your analysis to evaluate their stability using the NormFinder algorithm. You can also run the stability analysis with all genes. However, to avoid misinterpretation, it is recommended that a specific list of endogenous genes be informed. In our example, we included the Ct values of five endogenous genes: A (*ACTB*), B (*B2M*), C (*GAPDH*), D (*GUSB*), and E (*HPRT1*). So, we specify this gene list in the application (Figs. 4A, B) and request their evaluation. The system will retrieve the stability values of these genes and their combinations. We can then request to normalize our data with default parameters (Fig. 4C) to use the best combination of two genes. As a result, the system will display a message informing which genes will be used to normalize the gene expression data (genes B and E). At this point, all normalized data will be submitted for all subsequent modules. A table with the normalized -ΔCt values will be available for download on this tab (Fig. 4D).

### Expression analysis

**Fig. 5:**
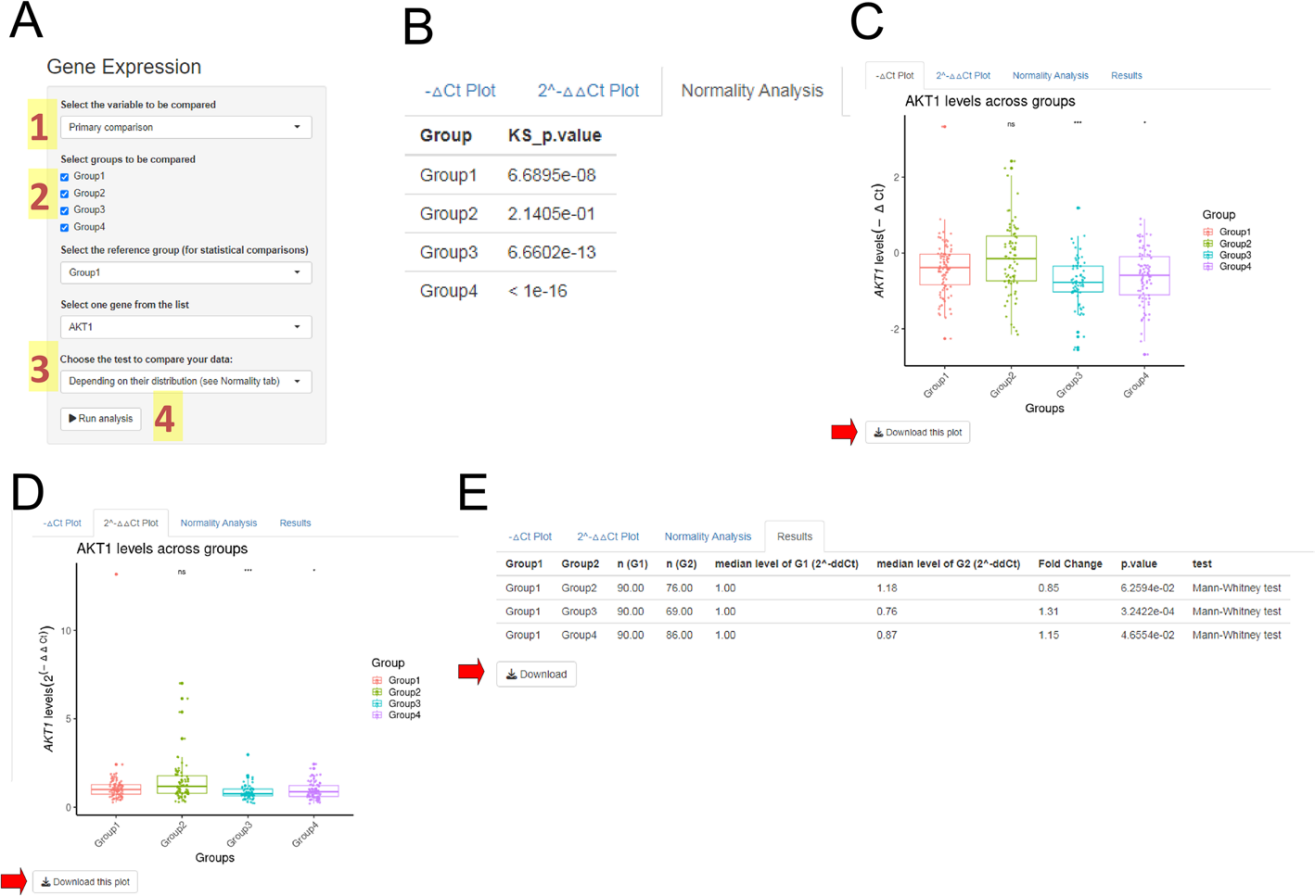
Expression Analysis module. This module automatically receives the normalized data and update the option questions with your data (**A**). To run the expression analysis, you should confirm the variable that will be compared (1) and define the groups that will be analyzed (including the reference group) and the gene of interest (2). You can choose to run a parametric or non-parametric test (3). However, it is suggested that the system make this choice based on your data distribution. After defining all the input information, you can run the analysis (4). This module will show a normality analysis of the gene expression for each group (**B**), boxplots showing −ΔCt (**C**) and 2^(−ΔΔCt)^ (**D**) data by primary groups, and a table summarizing all results generated for the selected gene (**E**). Red arrows show the respective buttons for downloading boxplots and the results table

After data normalization, all input fields of the Expression Analysis module will be filled with your data (gene names and group names; Fig. 5A). Then, you are requested to choose the groups that will be plotted, the reference group for statistical comparisons and calculation of 2^(−ΔΔCt)^ values, and the gene of interest. Next, you click on the "Run Analysis" button to run comparisons and display results. The system will automatically run the proper statistical analysis depending on your data distribution. After running the analysis, you can check the Normality Analysis tab for evaluation of the *p* values of the data distribution about gene expression levels in each group (Fig. 5B). Then boxplot graphs showing all participating samples will be displayed in the “−ΔCt Plot” (Fig. 5C) and “2^−ΔΔCt^ Plot” (Fig. 5D) tabs. These figures include statistical comparisons with the reference group using the asterisk format and can be downloaded in 600 dots per inch resolution (ready for publication). In addition, you can obtain a downloadable table showing all pairwise group comparisons (Fig. 5E) that includes median expression levels by group, fold change, *p* value, and method used. Moreover, users can review statistical variations by running parametric or no-parametric tests.

Optionally, if you load secondary information for your samples using a .xlsx (or .xls) spreadsheet, you can access these classifications by changing the “variable to be compared” in the input form (Fig. 5A).

In our example, we compared Group 1 against all other groups for *AKT1* gene expression levels. Once three of the four groups showed significant *p* values (*p* < 0.01) in the normal distribution test (Fig. 5B), the Mann–Whitney test (non-parametric) was applied to compare *AKT1* expression levels. Group 3 and Group 4 showed higher expression levels of the *AKT1* gene than Group 1 (*p* < 0.001 and *p* < 0.05, respectively; Fig. 5C–E).

### Volcano plots

**Fig. 6:**
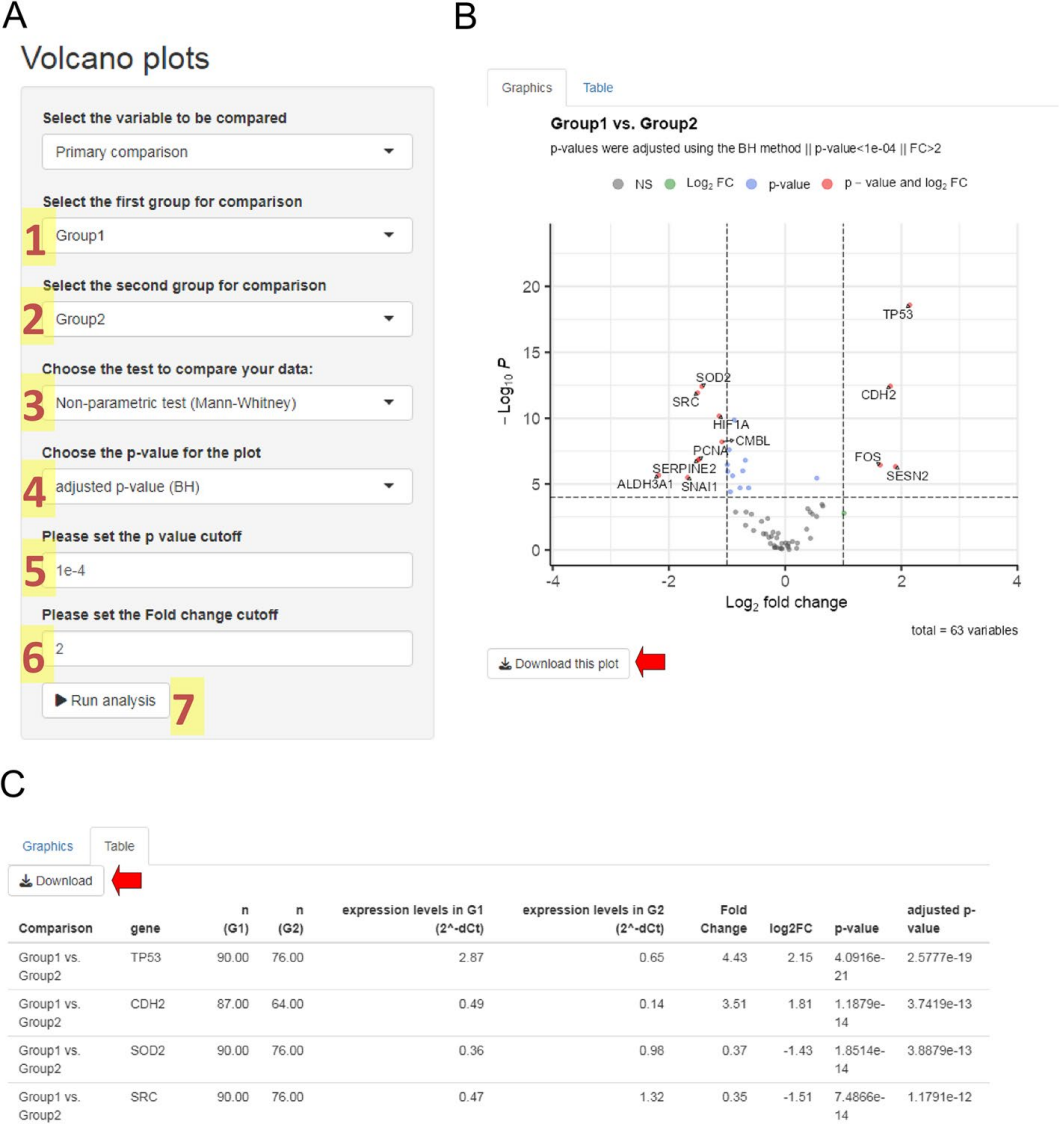
Volcano Plots module. (**A**) Input parameters required for the analysis. For this module, it is necessary to choose two different groups (1 and 2), define the type of analysis (3), set the type of *p* value to plot (4), and set the Fold Change (FC) (5) and *p* value (6) thresholds. Once all input information is defined, you can start the analysis (7). After estimating all fold changes and *p* values, the system will show the volcano plot (**B**) and the summary table (**C**). The volcano plot shows one point per analyzed gene, whose position depends on the log_2_ of its fold change and the -log_10_ *p* value. Considering the parameters set in panel A, the volcano plot shows labels only for all differentially expressed genes. A color legend is included to classify genes according to the fold change and *p* value levels. Red arrows show the respective buttons for downloading boxplots and the results table

For this module, we must select two groups for comparison of all gene expression levels. In the input panel, we can define parameters for the analysis and visualization of the volcano plots (Fig. 6A). For the analysis, the system requires two different groups and the type of test to be performed (Mann–Whitney or Student t-test). In our example, we analyzed our gene expression levels (−ΔCt) using the Mann–Whitney test between Group 1 and Group 2. For the volcano plot display, we can set the type of *p* value (adjusted or unadjusted) to plot and thresholds for fold change and *p* values. After running this module, the system displays the volcano plot, showing all differentially expressed genes between the selected groups (Fig. 6B). When the fold change (FC) between both groups and the *p* value exceeds the respective thresholds, a gene will be considered as differentially expressed. In our example, FC > 2 and *p* value < 1 × 10^–4^. In addition, the system also shows a table with all results for each gene: the performed test, gene name, number of samples in both groups, median expression levels in both groups, fold change, log_2_ of the fold change, *p* value, and adjusted *p* value (Fig. 6C). This module also allows downloading the plot and table in a publishable format. In our example, the comparison between Group 1 and Group 2 reports 12 differentially expressed genes. *TP53, CDH2, FOS*, and *SESN2* genes were upregulated in Group 1, whereas *SOD2, SRC, HIF1A, CMBL, PCNA, SERPINE2, ALDH3A1*, and *SNAI1* genes were upregulated in Group 2. In this module, you can also create volcano diagrams for secondary sample classifications once this information is successfully uploaded to the second sheet of the input file.

### Correlation analysis

**Fig. 7:**
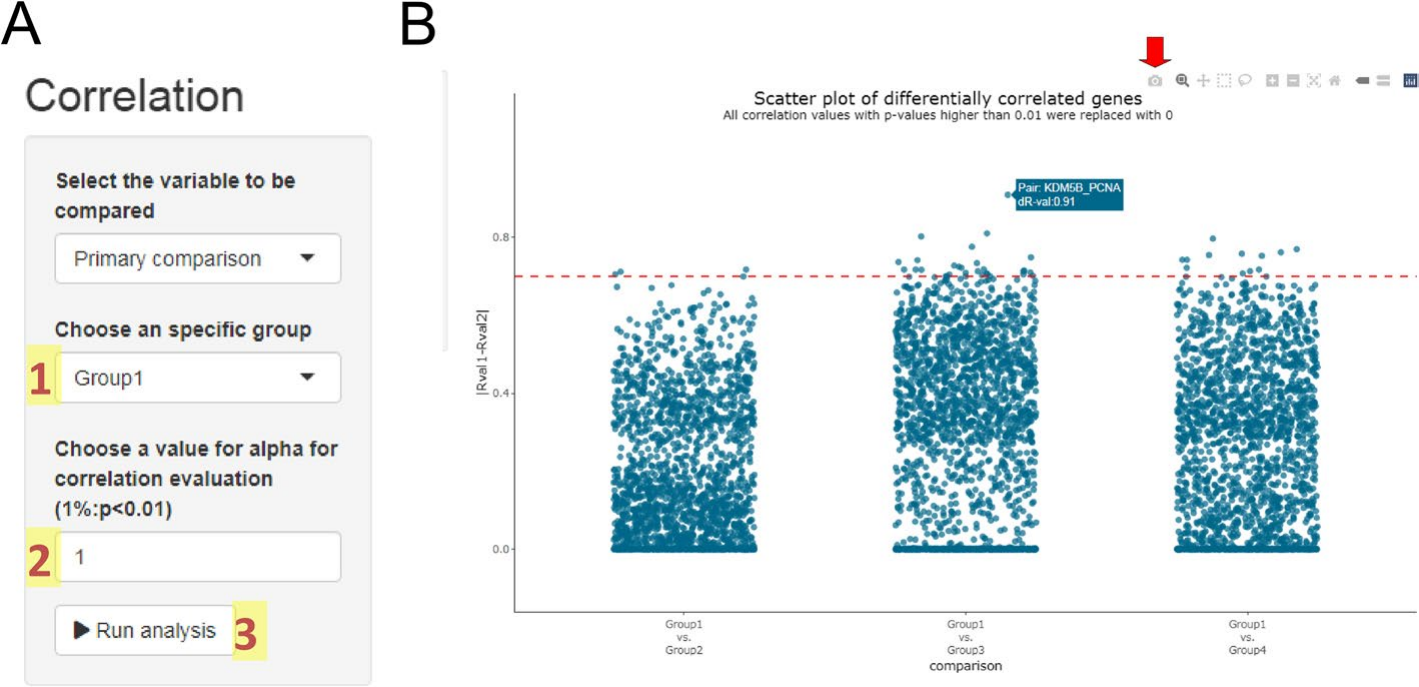
Correlation analysis module. This module will show the most differentially correlated gene pairs. To run it, we need to define the reference group (1) and the alpha value (2) in the setting panel (**A**). After defining all the input information, you can run the analysis (3). The system shows pairwise group comparisons, including the reference group, and will estimate the variation in correlation values for each pair of genes. Then, all values will be displayed in a dynamic plot (**B**). You can slice the mouse to get information for each pair of genes in each pairwise group comparison. Although this is not a plane figure, you can download a snapshot by clicking on the camera featured by the red arrow

This module evaluates differences in the correlation profiles of two genes between two groups. For that, we need to select a reference group and the alpha value (Fig. 7A). The analysis will show only the combinations in which the reference group participates. In our example, the dataset has four groups (Group 1, Group 2, Group 3, and Group 4). We then select Group 1 as the reference. So the system will show the variations in the correlation values of all gene pairs for the following pairwise comparisons: Group 1 vs. Group 2, Group 1 vs. Group 3, and Group 1 vs. Group 4. Then, the alpha value will be used to determine which correlations are considered statistically significant. All r-values with a *p* value higher than the alpha value will be converted to zero. After running the analysis, a dynamic dot plot will be displayed (Fig. 7B). Since the example dataset has four groups, three comparisons involving Group 1 (reference) will be shown. For each comparison, the number of plotted dots was determined by the number of pairwise gene combinations. Therefore, each point represents the variation in the correlation value for a pair of genes between the two groups in the analysis, the differential r-value (dR-val). Then, it is enough to slide the mouse over each point to observe the participating genes and the variation in the correlation values. In Fig. 7B, the pair involving *KDM5B* and *PCNA* genes shows a high variation rate equal to 0.91 between Group 1 and Group 3. Therefore, it may possibly represent the signature of some regulatory disruption between the two genes, which proposes itself as a new putative interaction/biomarker. Again, we can run this test for primary or secondary sample information based on its availability.

### Scatter plots

**Fig. 8:**
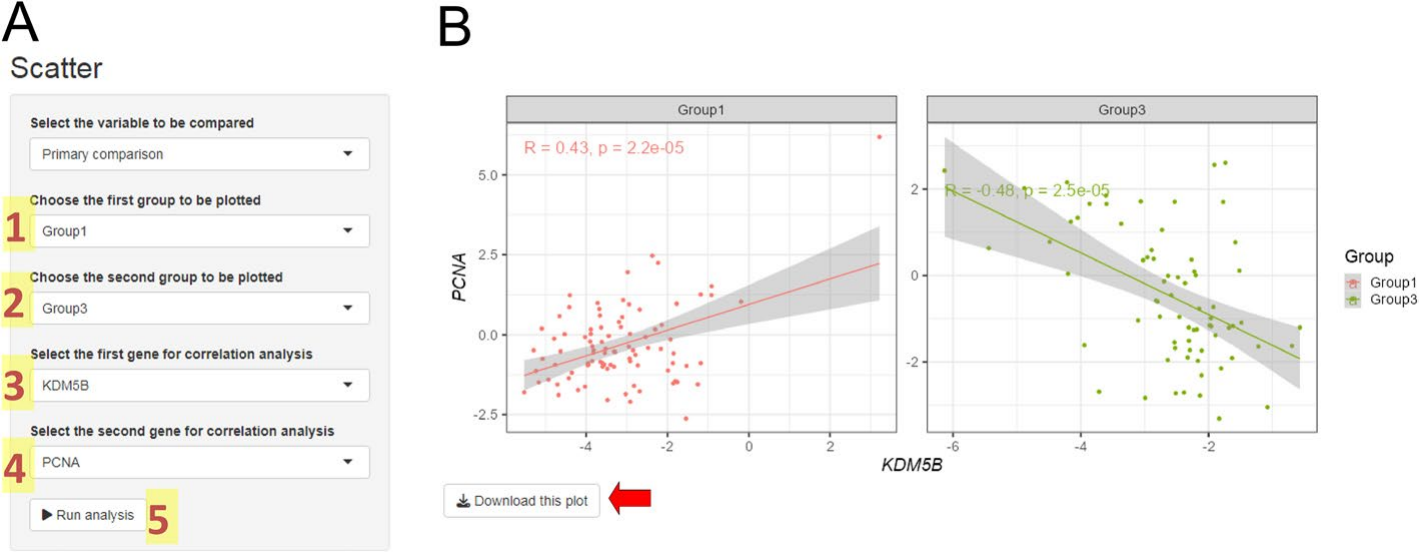
Scatter Plots module. This module allows us to confirm the findings of the correlation analysis module. Here, you must define in the input panel (**A**) two groups (1 and 2) and two genes (3 and 4) to be compared. Once you set all input information, you can run the analysis (5). The result will show two XY plots with one gene per axis (**B**). Each plot corresponds to a group and includes correlation features that reflect the variation observed in the previous module. The red arrow signs the button for downloading the figure in 600 dpi resolution

After we evaluate the differentially correlated pair of genes, we can validate these observations using the last analytical module. In this module, we need to inform the two groups and two genes to be shown. Following our example, we found that *KDM5B* and *PCNA* were differentially correlated between Group 1 and Group 3 (Fig. 7B). We will use these data to construct the scatter plot (Fig. 8A). After clicking in “Run Analysis” button, we see a two-panel figure (one panel per group) showing an XY plot with the representation of one gene per axis (Fig. 8B). Each panel includes a trend line and correlation features (r and p values). After screening data in the previous module, Fig. 8B shows the change in the correlation profile between *PCNA* and *KDM5B* genes in Group 1 and Group 3. It changes from a moderately positive correlation (r = 0.43) in Group 1 to a moderately negative correlation (r = − 0.48) in Group 3. In this module, we can also evaluate secondary information for the analyzed samples. You only need to choose the appropriate variable to analyze in the setting panel (Fig. 8A).

### Glossary

**Fig. 9:**
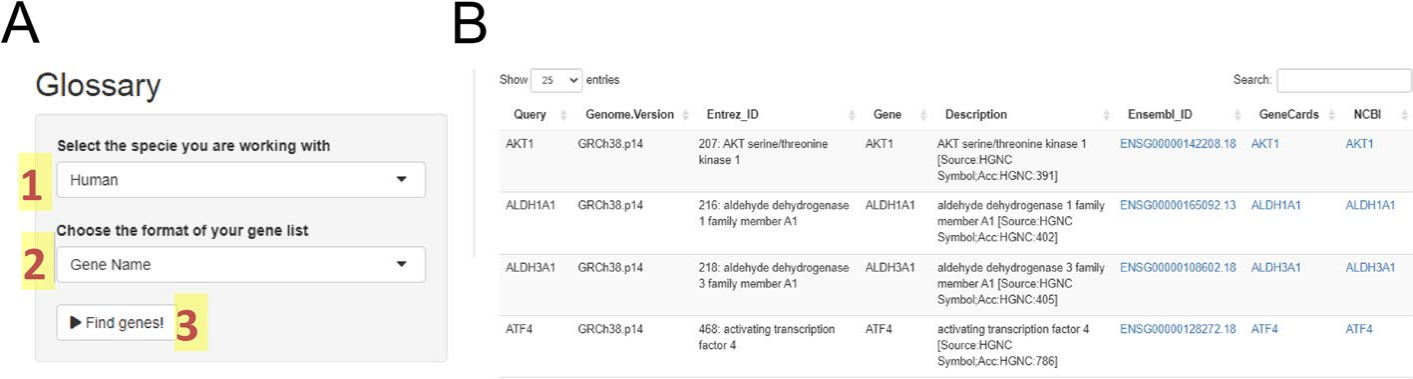
Glossary module. In this module, you only need to inform in the setting panel (**A**) the specie (1) and the format of your gene list (2) to search for it in the database (3). For all genes found in the database, you can see additional information and links to external sources (**B**)

This module allows us to get more information about the analyzed genes. Herein, you only need to specify the species (human, rat, mouse, or *D. melanogaster*) and the format of the gene list: Gene Name (e.g. "AKT1"), Entrez ID (p.e. 207), Ensembl Gene ID (p.e. "ENSG00000142208"), or Ensembl Transcript ID (p.e. "ENST00000649815"). After running this module (Fig. 9A), you can observe data from your gene list as an external gene name, Entrez ID, Ensembl ID, and external links to Ensembl, Genbank, and GeneCards databases (Fig. 9B). The app will also display the genome version of the Ensembl database that was loaded into the system.

## Discussion

Since there are different tools to analyze single-cell data generated by NGS [3, 22] but not many for qPCR [10–13], the ΔXpress application arises to analyze qPCR data on a large scale in a freely available, responsive, easy-to-use, reliable, and reproducible way. According to our records, some instruments have been developed to allow high-throughput data analysis by running single-cell or large-scale experiments by qPCR [7–9]. Although they have dedicated software for data preprocessing, that application software does not normalize or compare expression levels with categorical sample information, making our application unprecedented for this function in scalable qPCR-based experiments.

In addition, some academic web servers have been proposed to run qPCR data analyses, specifically for bulk essays. Table 1 summarizes web-based applications still available on line that allow users to analyze their qPCR data without requiring any software or expertise in additional programming languages. Herein, the ΔXpress app shares some functions with other web-based apps. For example, the Ct normalization by the Livak method and a user-friendly appearance provided by R and Shiny software. Nevertheless, the ΔXpress app also concentrated relevant functions to accept four different input formats, run a stability evaluation of housekeeping candidate regions before normalization, allow comparing more variables per run (using the same input file), automatically choose the proper test (parametric or no-parametric) for comparing your data according on their distribution, and allow downloading selected results for each run, which saves local memory and computational resources. All these functions are applicable for bulk, large-scale, or single-cell analyses. Moreover, only the ΔXpress app integrates four modules for advanced data visualization: Volcano Plots, Correlation Analysis, Scatter Plots, and Glossary. In particular, the Correlation Analysis and Scatter Plots modules allow us to envisage a single-cell approach to find differentially correlated genes.

**Table 1:** Comparison of principal features among currently available web-based applications for qPCR data analysis

Software	Accepted input formats	qPCR data normalization	Languages for web-based use	Support for more than one housekeeping region	Number of replicates allowed per sample	Variables allowed to be compared per run	Online visualization and customized download of the results	Additional functions	References
qRAT	.txt, .csv	Livak	back-end: R, front-end: HTML (free)	Yes. Arithmetic mean with no stability evaluation	Unlimited once identical for all samples	Only one variable	Yes	Filter by replicate variability and range of Ct, allow to download the normalized data and tabular results of analyses	[10]
Auto-qPCR	.txt, .csv	Livak	back-end: Python, front-end: HTML (free)	Yes. Arithmetic mean with no stability evaluation	Unlimited once identical for all samples	Up to two variables	No	Filter by replicate variability, allow to set the order of targets and samples, allow to download the normalized data	[11]
PIPE-T	.txt	Livak	back-end: R, front-end: HTML (inside Galaxy)	Yes. Arithmetic mean after stability evaluation (geNorm)	Unlimited, not required to be identical for all samples	Only one variable	No	Data imputation (cubic spline)	[12]
Shinycurves	.xls, .xlsx, .csv	Not specified	back-end: R, front-end: HTML (free)	No	Unlimited once identical for all samples	No variables allowed	Yes	Classification of samples as positive or negative (COVID-19 diagnosis), allow to download the normalized data and tabular results of analyses	[13]
ΔXpress	.xls, .xlsx, .txt, .csv	Livak	back-end: R, front-end: HTML (free)	Yes. Arithmetic mean after stability evaluation (Normfinder) or user-defined	Unlimited, not required to be identical for all samples	Unlimited variables (allowed for .xlsx or .xls files)	Yes	Generation of Volcano plots, analysis of differentially correlated genes,allow to download the normalized data and tabular results of analyses, annotate participating genes (Ensembl, NCBI)	This study

Finally, the exclusive Glossary module gives additional information for analyzed regions in an automatized way, representing a high differential compared with previously launched software.

Among additional functions observed in other software and absent in ΔXpress, the current version of our app cannot filter replicated samples by their variability or set a range of acceptable Ct values before running analyses. This is because single-cell experiments accept each cell-related expression value as one replicate, and the quality evaluation is performed after the lecture in the equipment, excluding dead cells, duplets, or triplets, as well as expression values with low reference quality.

Interestingly, the PIPE-T software allows users to impute missing data in analyzed datasets (Table 1). For the ΔXpress app, we excluded data imputation as we cannot control auxiliary variables required to reduce biased information in entered results [23, 24].

Naturally, as our application is provided using different R-packages and previously published algorithms such as NormFinder, we envisage their limitations as a source of improvement. Many of these limitations are related to the different input/output formats between these algorithms. To mitigate it, we developed additional code to make a built-in system that connects all these formats in a user-friendly environment.

As the ΔXpress application aims to map potential codependent regions through differentially correlated pairwise genes, this version of the app does not include unsupervised analyses such as heatmaps, principal component analysis (PCA), or t-Distributed Stochastic Neighbor Embedding (t-SNE). We restricted unsupervised functions for a second application, which will be open for a broad range of counts produced in omics-related experiments (genes, proteins, or metabolites). Ideally, we plan to connect both applications in an integrated analytical suite.

Also, this application was intentionally developed with restriction for statistical comparison between two groups because of two reasons. First, the strategy to find potential co-dependent genes or molecular switches of pathways is more reasonable in the analysis of pairwise groups as it has been previously shown in the ACHILLES project [25], a comprehensive atlas for co-dependent genes using RNAseq data. And second, the inclusion of tests for comparing means in multiple groups could add setting parameters reducing the practicality of our application. On the other hand, large-scale normalized data is double affected by variations of Ct values from the target gene and the housekeeping regions. Due to this reason, normalized expression data of qPCR experiments may not follow a normal distribution, which reinforces the use of median (instead of mean) and interquartile range (IQR, instead of standard deviation) for better representing their amplitude. Our app implements the use of medians and IQR as referential values per gene and group. In addition, the system evaluates the data distribution for each requested comparison and runs the proper statistical test (parametric or no-parametric).

Since qPCR can be used as an orthogonal or exploratory large-scale technique, we envision that the process for normalizing data and visualizing results should be user-friendly, reliable, and reproducible. However, we include relevant options to make each module customizable. It regards the most important feature to be controlled in each analysis (*p* value threshold, type of analysis, or control group). Then, in agreement with transparency-in-science principles, the results of our application (for example, volcano graphs) will include the parameters defined by the user. Additionally, all images produced by our system are downloadable in a ready-to-publication resolution (600 dots per inch).

The most impactful feature of our app is related to the portability of data and their analyses. By simply uploading a file in the appropriate format, researchers can elaborate tables and images without cluttering up their local storage. In our example, we loaded a table with data for 68 genes and 328 samples from four experimental groups. After normalizing the expression data of our target genes, we can download a table with –ΔCt values. We have chosen two housekeeping genes (out of five candidates) based on their stability across samples. Then, we can use the “Expression Analysis” module to produce over 500 analyses with their respective images. It includes observations for all 63 target genes, selecting one of the four groups as a reference and showing – ΔCt or 2^(−ΔΔCt)^ values. For the “Volcano Plots” module, we can use the same dataset for performing six analyses for all pairwise group combinations and display 12 volcano plots with nominal or adjusted *p* values. In the same way, the “Correlation Analysis” module produces at least four dynamic images for this data set. Subsequently, these images can help to filter the results of the “Scatter Plots” module. This filtering is necessary since the last section allows us to show pairwise combinations for groups (four in our example) and target genes (63 in our example), which results in almost 12 thousand images. Therefore, using our application, researchers can visualize their results only downloading what they need, work on different computers while preserving the code (and the results produced with their data), and reduce time consumption and local storage as this app does not require additional software to be installed.

**Fig. 10:**
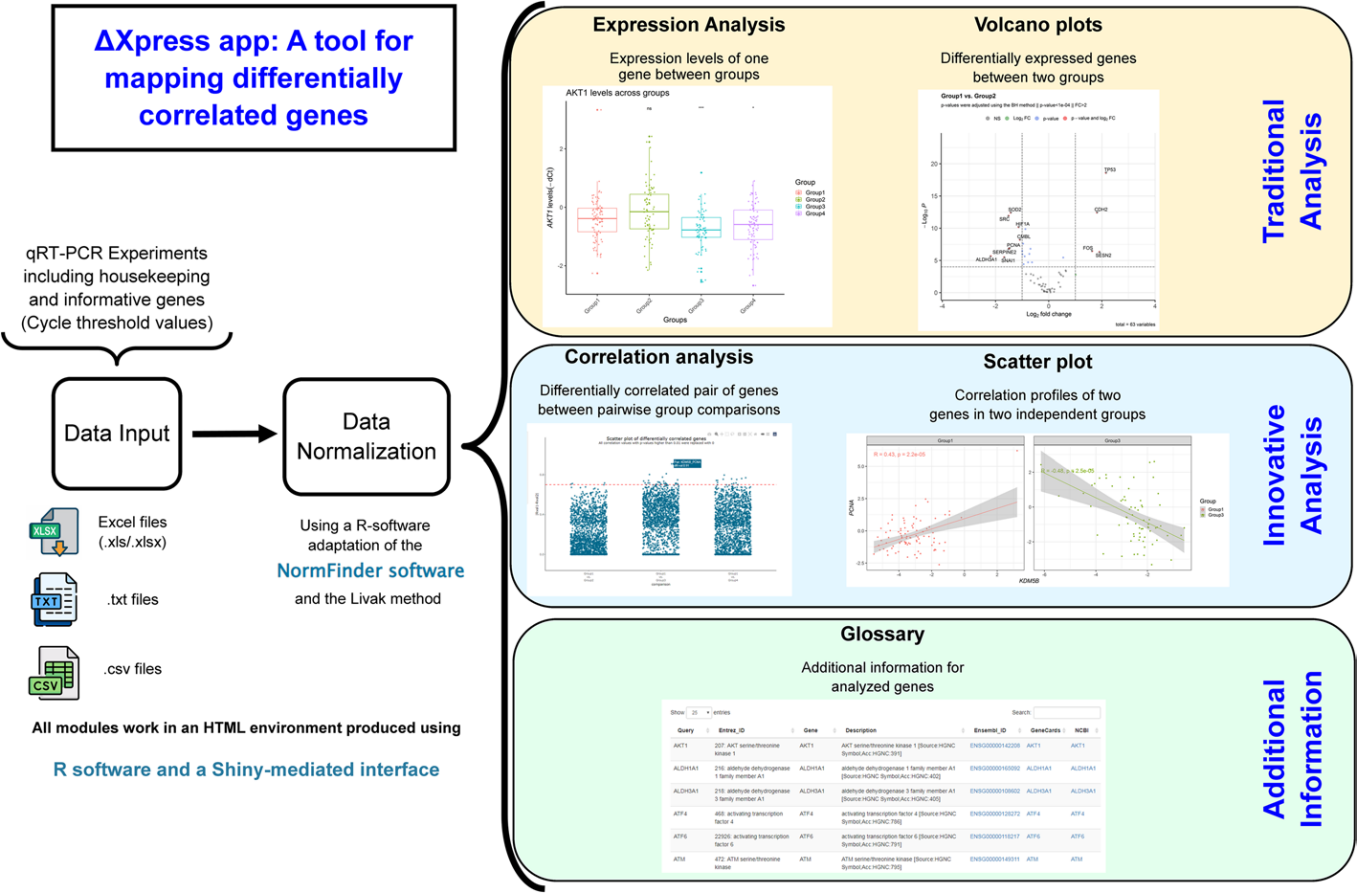
Visual abstract of the ΔXpress application

Currently, the Glossary is limited to a few species (*Homo sapiens*, *Drosophila melanogaster*, *Mus musculus*, and *Rattus norvegicus*), but we can add other species upon request. All the other modules can be used for all biological data potentially available for qPCR experiments. For instance, our research has currently used this application looking for differentially expressed genes between groups and also for mapping differentially correlated genes in a cancer environment. As a summary of functions and main characteristics of the ΔXpress application, we elaborated a visual abstract in Fig. 10.

## Conclusions

We developed the ΔXpress application to analyze single-cell or large-scale qPCR data from normalization to conventional gene expression analysis and differentially correlated gene mapping to add information about potential disruptions in context-dependent experiments, producing also ready-to-publication images. The application is available on the Shinyapps web server (https://alexismurillo.shinyapps.io/dXpress/) for non-commercial purposes.

## Availability and requirements

Project name: deltaXpress (ΔXpress) app

Project home page: https://alexismurillo.shinyapps.io/dXpress/

Operating system(s): Platform independent

Programming language: R and HTML

Other requirements: Updated browser chosen by the user

License: GNU GPL-v3

45Any restrictions to use by non-academics: None

### Supplementary Information

**Additional file 1.** Example files for the ΔXpress app. Zipped folder containing example data in three file formats (.csv, .txt, and .xlsx).

## Data Availability

The ΔXpress app is available online at https://alexismurillo.shinyapps.io/dXpress/. A zipped folder with three example files (different input formats) was attached as Additional file 1, and it is also available with the source code at https://github.com/Murillo22/dXpress.

## Abbreviations

FC: Fold change
IQR: Interquartile range
qPCR: Quantitative polymerase chain reaction
Ct: Cycle threshold
NGS: Next generation sequencing
dR-val: Differential R-value
PCA: Principal component analysis
t-SNE: T-distributed stochastic neighbor embedding
